# A Web-Based Physical Activity Portal for Individuals Living With a Spinal Cord Injury: Qualitative Study

**DOI:** 10.2196/12507

**Published:** 2019-07-26

**Authors:** Max Pancer, Melissa Manganaro, Isabella Pace, Patrick Marion, Dany H Gagnon, Marie-Thérèse Laramée, Frédéric Messier, Fatima Amari, Sara Ahmed

**Affiliations:** 1 School of Physical & Occupational Therapy Faculty of Medicine McGill University Montreal, QC Canada; 2 Center for Interdisciplinary Research in Rehabilitation of Greater Montreal Montreal, QC Canada; 3 École de Réadaptation Université de Montréal Montreal, QC Canada; 4 Centre Intégré Universitaire de Santé et de Services Sociaux de l’Est-de-l’Île-de-Montréal - Institut Universitaire sur la Réadaptation en Déficience Physique de Montréal Montreal, QC Canada; 5 Centres Intégrés Universitaires de Santé et de Services Sociaux du Centre-Ouest-de-l’Île-de-Montréal Montreal, QC Canada; 6 McGill University Health Center Montreal, QC Canada

**Keywords:** spinal cord injuries, self-management, internet, exercise, motivation, volition

## Abstract

**Background:**

The population with a spinal cord injury (SCI) largely remains inactive following discharge from rehabilitation despite evidence on the benefits of physical activity. These individuals need to develop skills to self-manage their condition in order to prevent secondary comorbidities and rehospitalization. A Web-based physical activity portal can address this need. Few Web-based interventions incorporate theoretical frameworks, behavior change techniques, and modes of delivery into their design.

**Objective:**

This study aimed to identify the preferred features of a Web-based self-management physical activity portal through stakeholder engagement with individuals with a spinal cord injury and health care professionals (HCPs).

**Methods:**

An interpretative phenomenology methodology and participatory design, along with an integrated knowledge translation approach, were used to conduct this study. Convenience sampling was used to recruit individuals with an SCI living in the community, who were either interested or already engaging in physical activity, and HCPs working with individuals with an SCI, from three city-based rehabilitation sites. Individual 1-hour sessions involving navigation of an existing website and a semistructured interview were conducted with all participants. Individuals with an SCI completed a demographics questionnaire prior to the individual sessions, while demographic information of the HCPs was collected during their interviews. Additionally, all participants were asked a question on their intention to use or recommend a portal. An in-depth thematic analysis was used to derive themes from participants’ responses.

**Results:**

Thirteen individuals with an SCI and nine HCPs participated in the study. Five core themes emerged: (1) knowledge: guidance and barrier management; (2) possibility of achievement: the risks and benefits of physical activity and modelling; (3) self-regulation strategies: action planning, goal setting, tracking, rewards, and reminders; (4) interactivity: peers and professionals; and (5) format: appearance, language, and ease of use. The mean (median) ratings of the likelihood of promoting and using a Web-based portal tailored to individuals’ needs were 9.00 (8.78) and 7.75 (7.88) for HCPs and individuals with an SCI, respectively.

**Conclusions:**

This study highlights features of an online self-management platform that can provide individuals with an SCI the motivation and volition to engage in physical activity. These findings will inform the design of a Web-based self-management physical activity portal to increase physical activity adherence and behavior change.

## Introduction

Persons living with a chronic disability, especially those with limited mobility, are at an increased risk of developing secondary comorbidities, many of which are preventable [1]. With the rise in the prevalence of these conditions, individuals are expected to effectively manage their condition to ensure a high quality of life and longevity [2-5]. One response to this concern is the increasing development of self-management systems, specifically those that are Web-based. Due to continual technological advances, the internet has changed the way in which individuals seek and receive health services and information [6]. There are now a variety of online resources that have the potential to support health and well-being. Moreover, research has reported increased levels of condition-related information searching among chronic disease populations, including diabetes, depression, and chronic obstructive pulmonary disorder (COPD) [7,8]. These technologies have been recognized as a vital component for adherence to chronic disease management and the prevention of secondary health concerns [1,3,5].

Although great variability exists, Web-based self-management systems largely include components addressing multiple facets of disease management [9]. One major area of concern that can have a significant impact on medical, symptom, and lifestyle management is physical activity. In fact, the World Health Organization and the Center for Disease Control and Prevention have described physical inactivity as a leading risk factor for chronic conditions and death [10]. Conversely, regular physical activity has been shown to decrease the development of secondary comorbidities [10]. In effect, there is a need for health promotion efforts pertaining to physical activity engagement among individuals living with a chronic condition [1].

A spinal cord injury (SCI) is one such condition that requires comprehensive self-management interventions to improve the quality of life of members of this population. Individuals with an SCI are discharged from rehabilitation hospitals following increasingly shorter lengths of stay. The SCI population largely remains physically inactive after discharge, despite available physical activity guidelines and knowledge of the benefits of physical activity [11-13]. As seen in other chronic conditions [5], these individuals acquire fewer skills to self-manage their condition upon reintegration into the community, as information regarding their injury becomes less accessible [14,15]. In their systematic review of meta-analyses, Rogers et al [16] found that most of the self-management programs on the internet that demonstrated effectiveness in randomized controlled trials are unavailable for use to the general public. Therefore, these individuals are at an increased risk of developing secondary comorbidities, such as autonomic dysfunction, loss of motor control, depression, and fatigue [12,17], often resulting in rehospitalization [15]. To address this inactivity and the development of secondary health conditions, knowledge must be mobilized beyond academics and into the hands of individuals living with chronic conditions via optimal delivery mechanisms to enhance information uptake [12,18].

Additionally, the vast majority of reviews that evaluate the effectiveness of Web-based self-management interventions have focused on chronic conditions, specifically, those with a more progressive nature such as obesity, type 2 diabetes, and COPD [4,8,19]. Although physical activity is undoubtedly important for individuals with and those without disability, the SCI population presents with a unique need for support and resources, as the rapid, often immediate, onset of an SCI necessitates intense rehabilitation services early on and a high level of resilience to cope. In effect, there is a need for novel research that systematically explores and describes effective technologies to be included in an online self-management program for individuals with an SCI and other populations with similar needs. Furthermore, only a small number of available Web-based interventions are grounded in theoretical frameworks and incorporate behavior change techniques (BCTs) and modes of delivery (MoDs) into their design [20-22]. Theories are valuable in understanding the factors that influence one’s health behaviors [23]. Additionally, incorporating BCTs and MoDs into the design of online self-management programs can inform adherence and behavior change [24-28].

The Health Action Process Approach (HAPA) is a framework that effectively addresses the intention-behavior gap [29] and has been applied in rehabilitation with clinical populations [23]. It posits that individuals require *motivation* to develop the intention to engage in a behavior, followed by *volition* to elicit a behavior [29]. It proposes that characteristics of Web-based interventions, including BCTs and MoDs, should be adapted to one’s stage of readiness to engage in a behavior. Thus, the framework provides a theoretical foundation to guide the development of self-management programs to increase adherence behaviors [30]. It has demonstrated good applicability in increasing adherence to physical activity among individuals with obesity and type 2 diabetes [19,23,31,32] as well as in creating physical activity interventions for individuals with an SCI [33,34].

According to the HAPA framework*, goal setting*, *action planning*, *provision of feedback on performance*, and *self-monitoring* are among the most effective BCTs linked to changes in health-related behavior [24-26]. The BCTs *barrier identification/problem solving* and *providing rewards on behavior change* have also been correlated with improvements in clinical and psychological outcomes as well as health behavior [27]. In terms of MoDs, *access to an advisor* /*contact with clinicians* was found to have small-to-medium effects on behavior [32] and has been linked to intervention adherence [26,28]. A thematic synthesis of qualitative studies by Dwarswaard et al [2] showed that individuals with chronic conditions require support from health care professionals (HCPs) to manage their condition. The collaboration between patients and HCPs is vital for self-management support [2]. Alternately, several studies have postulated that high attrition rates and minimal health behavior change during Web-based health-related programs may be associated with minimal or no contact with experts [35,36].

*SCI Action Canada* is an Ontario-based online self-management physical activity portal for individuals with an SCI, created in 2008 [37]. This website was developed to mobilize strategies to inform, teach, and enable individuals to initiate and maintain a physically active lifestyle [38]. It comprises various BCTs in line with the Health Action Process Approach framework [33,39] as well as multiple MoDs [38]. *SCI Action Canada* ’s content includes evidence-based physical activity guidelines, education on the benefits and safety precautions related to physical activity, tailored exercises and information based on the level of injury, and strategies to plan physical activity and overcome potential barriers. However, certain interactive features such as the possibility to contact a clinician as well as select customizable features that the user can individualize to their current condition and preferences are not incorporated in the website. Despite the evidence presented on the effectiveness of BCTs and MoDs in self-management portals, a knowledge gap remains regarding which Web-based features best meet the unique needs of individuals with an SCI and potentially other populations with shared characteristics and recovery trajectories.

Moreover, few studies have embedded a participatory or user-centered design that incorporates the perspectives of both HCPs and individuals with an SCI in the initial development of such a site [40,41]. This iterative research design constitutes a collaborative approach in which individuals who can impact or be impacted by an intervention are involved throughout its development. The inclusion of end-users in initial designs of a Web-based portal has been shown to enhance usability, adherence, and behavior change associated with its use [42]. Jafari et al [43] found that a participatory design approach was essential in determining the aspects needed in the creation of personalized, internet-enabled education for patients with diabetes. Furthermore, a participatory design study by Allin et al [44] demonstrated that engaging users as codesigners, codevelopers, and informants in the formation of an online platform may promote self-management.

In their study, Munce et al [15] used a cross-sectional design to explore preferred content modules, including exercise, pain, and nutrition management, as well as program delivery formats including the internet, DVDs, and a telehealth system to include in a program for individuals with an SCI. They suggested the creation of a tailored self-management program to increase users’ knowledge acquisition postdischarge from rehabilitation; their study was the first to examine these concepts using feedback from individuals with an SCI. This research study builds on Munce and colleagues’ [15] novel findings by examining specific Web-based components of a self-management portal focused on physical activity.

To our knowledge, this study is the first to explore the needs, learning styles, and preferences of key stakeholders, including individuals with an SCI and HCPs, specifically in the development of a Web-based self-management program. Thus, the objective of this study is to identify needs and preferences of individuals with an SCI and HCPs with regard to the features of a Web-based self-management physical activity portal that have the potential to enhance one’s motivation and volition to engage in physical activity.

## Methods

### Statement of Ethics

Approval for this project was granted by the Centre for Interdisciplinary Research in Rehabilitation of Greater Montreal. We certify that all applicable institutional and governmental regulations concerning the ethical use of human volunteers were followed during this research study.

### Research Design

This qualitative study used a constructivist paradigm with an interpretive phenomenology methodology, and an integrated knowledge translation approach to inform the objective of this research. This approach acknowledges the unique life experiences of individuals with a disability as well as the clinical experience of HCPs. It additionally permits an exploration through the analysis of first-person narratives constituting participants’ experiences, views, and needs. An in-depth content analysis was subsequently used to systematically determine participants’ preferred design features of an online physical activity platform. A participatory or user-centered design, which supports cocreation through the involvement of key stakeholders, was also applied.

### Recruitment

Convenience sampling was used to recruit individuals with an SCI and HCPs. Recruitment took place at three rehabilitation sites in the province of Quebec: Rehabilitation Site 1 was a Montreal-based secondary inpatient and outpatient rehabilitation hospital, Rehabilitation Site 2 was a Montreal-based tertiary inpatient and outpatient rehabilitation hospital, and Rehabilitation Site 3 was a Quebec City–based secondary and tertiary inpatient and outpatient rehabilitation hospital with postdischarge physical activity programs.

Recruitment of individuals with an SCI was done via the telephone. The researchers contacted interested persons with an SCI from Montreal and Quebec City. The eligibility criteria were as follows: age ≥18 years, discharged from inpatient services from one of the three rehabilitation sites, ability to communicate in English or French, ability to independently make informed decisions, access to the internet and an email address, and ability to use a computer independently or with assistance.

HCPs were recruited from the affiliated institutions through presentations to clinical teams to explain the objective of the study and the required time to participate. Interested HCPs contacted the researchers via email.

### Procedure

After obtaining informed consent, two trained researchers conducted individual 1-hour sessions with HCPs and individuals with an SCI at the individual’s affiliated rehabilitation site or in their home. Prior to their scheduled session, the individuals completed a demographics questionnaire.

In the first part of the session, participants spent 30 minutes navigating selected content on the *SCI Action Canada* website. Using a think-aloud approach, participants verbally expressed their immediate positive and negative thoughts on how the content and structure of the website may influence the motivation and volition of persons with an SCI to engage in physical activity. The two researchers tracked the content that participants independently navigated through, with the use of a comprehensive checklist containing the BCTs and MoDs integrated in the website. They also prompted participants to navigate through the features they overlooked. The website navigation session was recorded using Camtasia software, version 8 (TechSmith, Okemos, MI), provided by McGill University.

Subsequently, the two researchers conducted semistructured interviews comprised of open-ended questions during the second part of the session. Participants responded to questions regarding physical activity engagement following an SCI. Individuals with an SCI were asked about the type, frequency, and social aspect of, as well as barriers to, their involvement in physical activity since their injury. HCPs were asked about their experience with physical activity prescription for their patients and expounded on strategies to improve physical activity adherence within the SCI community. All participants explored the importance of physical activity for individuals with an SCI. They were also asked to provide feedback on the website navigation portion of the session, including the site’s ease of use, appeal, and delivery format, as well as perceived usefulness of embedded and absent physical activity resources. Furthermore, the individuals were asked to rate their likelihood of integrating a Web-based portal into their physical activity practice, on a scale of 0 (unlikely) to 10 (very likely). Using a similar scale, HCPs were asked to rate the degree to which they would promote such a physical activity platform in practice. All interviews were audio recorded using Camtasia software.

The length of each session ranged from 46 to 90 minutes. All sessions were transcribed verbatim. Pseudonyms were assigned to each participant. Quotations from French-speaking participants were translated to English for the purpose of this paper. In addition, the demographics questionnaire, interview guide, and navigation checklist used throughout the study were reviewed by a team of stakeholders, which included three clinical researchers, a knowledge translation specialist from *SCI Action Canada*, a coordinator for a Quebec adapted sports organization, and a consultant living with an SCI who promotes community integration.

### Data Analysis

Descriptive statistics were used to summarize the characteristics of all participants and the responses on questionnaires for individuals with an SCI. All transcripts from the site navigation and interview sessions were reviewed. An in-depth thematic analysis was conducted by four members of the research team to derive common themes. Content analysis was guided by participants’ rich feedback on the perceived ability of select Web-based features to impact the motivation of the individuals to initiate and maintain engagement in physical activity. The two authors who conducted the interviews also maintained field notes with thematic logging of significant moments during and after the individual sessions, which served as a component of data triangulation to contribute to the analytical process. Codes representing core concepts were identified in the transcripts. Through extensive discussion surrounding emerging commonalities, subthemes were formed and then grouped under “umbrella” key themes. All derived themes have connections to BCTs and MoDs, as outlined by Abraham and Michie [45]; this was paramount to contribute to the evidence and overall rhetoric surrounding their value as embedded components of Web-based self-management portals aimed at ameliorating health-related behaviors. The four researchers who carried out the analysis met regularly to resolve any discrepancies in order to ensure consistency of the findings. Data saturation was achieved at 21 participants. The median and mean ratings attributed to the likelihood of integrating a Web-based portal into physical activity practice or promotion were calculated.

## Results

### Participant Characteristics

Thirteen persons with an SCI, seven from Montreal and six from Quebec City, agreed to participate in the study. Ultimately, data collected from 12 individuals were included in the study, as data from one participant, whose condition turned out to be inaccurate, were removed. The majority of the sample was male (n=10) between the ages of 19 and 68 (mean 46.8, SD 17.1) years. Most of these individuals had a nontraumatic SCI (n=8) and a level of injury located between T1-S5 (n=6). Most participants were discharged from rehabilitation for more than 12 weeks (n=9). Ten of the participants had been participating in physical activity for over 6 months. Lastly, most subjects spent at least 2 hours per day navigating the internet, in general (n=8). Demographic information of these individuals is presented in Table 1.

Nine HCPs, six from Montreal and three from Quebec City, agreed to participate in the study. They had an average of 13.7 (SD 11.9) years of experience working with the SCI population. Additional demographic information of the HCPs can be found in Table 2.

In total, 5 themes with 14 subthemes emerged (Textbox 1).

**Table 1:** Characteristics of the individuals with a spinal cord injury.

Characteristic		n (%)
**Age (years)**		
	16-30	2 (17)
	31-45	2 (17)
	46-60	5 (42)
	61-75	3 (25)
**Gender**		
	Male	10 (83)
	Female	2 (17)
**Affiliated rehabilitation site(s)^a^**		
	Site 1	6 (50)
	Site 2	4 (33)
	Site 3	6 (50)
**Level of injury**		
	C1-C4	3 (25)
	C5-C8	3 (25)
	T1-S5	6 (50)
**Severity of injury**		
	Complete	5 (42)
	Incomplete	7 (58)
**Nature of injury**		
	Traumatic	4 (33)
	Nontraumatic	8 (67)
**Time since discharge from inpatient rehabilitation services (weeks)**		
	0-4	1 (8)
	5-8	1 (8)
	9-12	1 (8)
	>12	9 (75)
**Number of months participant engaged in physical activity**		
	≤6	2 (17)
	>6	10 (83)
**Hours per day** **participant** **spent on the internet, in general**		
	<1	1 (8)
	1 to <2	3 (25)
	2 to <3	5 (47)
	3 to <4	1 (8)
	>4	2 (17)

^a^Total N>12, as several participants attended more than one rehabilitation site.

**Table 2:** Characteristics of the health care professionals.

Characteristic		n (%)	
**Profession**			
	Physiotherapist		3 (33)
	Occupational therapist		3 (33)
	Kinesiologist		1 (11)
	Physical rehabilitation therapist		1 (11)
	Dietician/nutritionist		1 (11)
**Gender**			
	Male		2 (22)
	Female		7 (78)
**Affiliated rehabilitation site**			
	Site 1		3 (33)
	Site 2		3 (33)
	Site 3		3 (33)
**Years of work experience with the spinal cord injury population**			
	1-10		4 (44)
	11-20		2 (22)
	21-30		2 (22)
	>30		1 (11)

**Characteristic of Web-based interventions, themes, and subthemes.**

**Behavior change techniques**

- **Knowledge**
  - Guidance
  - Barrier management
- **Possibility of achievement**
  - Risks and benefits
  - Models
- **Self-regulation**
  - Action planning
  - Goal setting
  - Tracking
  - Rewards
  - Reminders

**Modes of delivery**

- **Interactivity**
  - Peer
  - Professional
- **Format**
  - Appearance
  - Language
  - Ease of use

#### Knowledge

Participants preferred resources that enhance their knowledge of physical activity in terms of guidance and barrier management.

##### Guidance

Participants identified the importance of including resources that guide them in their physical activity participation. These resources consisted of home-based exercise tutorials, safety suggestions, and evidence-based physical activity guidelines. Furthermore, participants preferred having access to practical materials from a reliable source.

Oh I like this(home-based exercise tutorials). This tells you how to do it, right? I like that. You know why I like that? Cause it’s stuff I can do at home and I don’t need anybody.

ISCI 13, female, 52 years

It’s important to engage in physical activity, but it must be done safely. People often fail to consider certain aspects of physical activity.

HCP 05, female, occupational therapist, rehabilitation site 2

##### Barrier Management

Participants identified cost and availability of exercise equipment, and physical accessibility as barriers to their physical activity participation. They suggested having access to information on tangible solutions to overcome these barriers.

That’s interesting, you know, (information on) overcoming obstacles because people often tell me “Ah! But you're training, you're lucky, you're moving your abdominals, you're moving your legs, and this and that” ...People have difficulty overcoming obstacles because there are no two people with the same disability, so it can be helpful to provide information on overcoming obstacles.

ISCI 10, male, 31 years

You know, the equipment, notably adapted bicycles, is so expensive, so, [access to equipment] can be difficult without financial support. Over here [RS3], we have resources that can lend adapted bicycles, a cycling club...there are several resources, but they need to be advertised.

HCP 07, female, occupational therapist, rehabilitation site 3

#### Possibility of Achievement

All participants, especially persons with an SCI, suggested including features that will inspire them to achieve their physical activity goals.

##### Risks and Benefits

Participants disclosed the importance of being aware of the benefits of physical activity participation and the negative consequences of remaining sedentary.

I think there should be more information on the benefits of physical activity...or the risks associated with not engaging in physical activity. You know, a little like what they do with cigarette packages: “If you smoke, you will develop this disease”, so on the site [it would say] “if you don’t move, you will become overweight, you will experience shoulder pain, you will end up in a power wheelchair and become dependent on others”. So, maybe not to say it this explicitly because it may offend certain users, but to really explain the benefits of physical activity and risks of a sedentary lifestyle.

ISCI 10, male, 31 years

Although I think [risks and benefits] can be motivating, they must be clearly outlined: you know “Essentially, physical activity improves this, and this, and decreases this” ...and also to explain that individual with an SCI are more at risk of developing other types of health problems and must be extra careful. Physical activity certainly helps maintain a healthy body weight, it can reduce risks of diabetes, it can reduce several things, so it’s very important.

HCP 05, female, occupational therapist, rehabilitation site 2

##### Modelling

Examples of individuals with an SCI successfully engaging in physical activity within a community setting, in the form of photos, testimonials, and a mentor, was a recurrent topic. The majority of the participants explained that these forms of modelling provide a tangible outlook on a physically active lifestyle.

When I see this photo [individuals with aN SCI participating in an exercise class], I’d like to do it like him. It’s very good...it [would] make me so happy. I want to do it like him.

ISCI 02, female, 26 years

Providing examples of active [individuals with an SCI] in the community, like success stories or testimonies or [saying] where individuals started and where they are now, and the benefits they have acquired...often speaks to clients; it gives them an idea, a vision of the future and of what it looks like to be an active person with an SCI.

HCP 06, female, kinesiologist, rehabilitation site 2

#### Self-Regulation Strategies

Many participants conveyed a preference for self-regulation tools such as action plans, goal setting and tracking, rewards, and reminder systems to support their engagement in physical activity.

##### Action Planning

Participants expressed that an action plan—a detailed organization of when, where, and how physical activity can be incorporated into one’s weekly schedule—provides a sense of structure.

I personally have an action plan embedded within my schedule because it is as important as medical appointments...To reinforce, to integrate [physical activity] in a calendar is super pertinent.

ISCI 12, male, 53 years

On the other hand, a number of the individuals stated that their action plan did not need to be written down or input on their phone or computer, as it was already ingrained in their lifestyle.

My schedule is in my head. The definition says it: it’s a routine. it’s over and over again.

ISCI 03, male, 43 years

##### Goal Setting

Participants desired a goal-setting system as an interactive Web-based feature to formulate specific, meaningful, and realistic physical activity objectives that follow a safe progression.

[A goal setting system] gives importance to physical activity. [Just like] organizing a trip, giving time to organize my things, it gives physical activity the same importance as these things.

ISCI 12, male, 53 years

[Gradually progressing goals] is important because when objectives are not progressive, people are intimidated, and they get injured, and finally they abandon it because the way they started was not necessarily realistic.

ISCI 07, male, 63 years

##### Tracking

Participants discussed the importance of having a tool that monitors their physical activity progress. Participants reported that this tool would enable them to measure their improvements and follow-up on their objectives in order to stay accountable to their physical activity plan.

It would be very interesting to access the history of your past physical activities and exercise programs, to see if there has been progress

ISCI 05, male, 59 years

[To follow] the physical activity you have accomplished in real time, to know what you have accomplished and what is left to accomplish a given objective, I always think of a circle that becomes filled in as you complete more and more hours of physical activity.

ISCI 09, male, 19 years

##### Rewards

Some of the individuals with an SCI favored incorporating a reward system on a website to reinforce the habit of engaging in physical activity. These individuals reported that they enjoy rewarding themselves for engaging in physical activity with a positive extrinsic stimulus.

If I do my full 40(swimming)laps, I get a piece of cake. If I don’t, I don’t get it. That’s me...Because I’m telling you, when I’m really tired and I don’t want to do the 40 laps and I think of that piece of cake, I’m going to go [do the 40 laps].

ISCI 13, female, 52 years

However, some participants did not agree with the idea of a reward system, as they felt the health benefits of physical activity are a sufficient reward.

When you participate in physical activity, you do it for yourself. Your [reward], you will get it when you reap the benefits...Improving your condition is the best reward you can get.

ISCI 07, male, 63 years

All the benefits it can bring, maintaining a healthy weight, energy levels, decreasing stress, that is what’s important to me. I think the individual who wants to be active has to do it for themselves and not for a reward system.

HCP 07, female, occupational therapist, rehabilitation site 3

##### Reminders

Some of the individuals explained that having a reminder system to participate in physical activity can serve as a form of encouragement or compensate for memory difficulties.

It’s good. It will make me, maybe feel like somebody is pushing me to work; yeah, it’s good. Like support.

ISCI 02, female, 26 years

However, others suggested that this type of system should be optional, as reminders can become a stressor for them.

Personally, I hate it. I hate it! It's like...I think we have so much pressure from everything else. I wouldn’t like to have reminders, but I’m sure some people would like it.

ISCI 05, male, 59 years

#### Interactivity

Participants highlighted the value of having access to features that enable interaction with peers and professionals, as it would provide a sense of support.

##### Peer

Participants reported that they would value platforms to share resources and anecdotes, such as discussion forums or chat groups, which provide guidance and help foster a sense of belonging.

It (a network) is super important. If you don’t have one, you don’t have much. I often say “If i had not met all these people (with an SCI), I would be isolated and depressed,” so it’s super important.

ISCI 09, male, 19 years

It could be a forum with questions, but also a forum where they can exchange information: “I would like to start a basketball team, but I am missing participants. Who would be available?”

HCP 05, female, occupational therapist, rehabilitation site 2

##### Professional

Persons with an SCI expressed the importance of having access to a HCP by phone, email, or Skype, following discharge from rehabilitation, as it would enable them to seek reassurance with their physical activity plan or answer any questions they may have. Ultimately, they reported that having this type of access contributes to a continuum of care upon discharge.

It’s an alternative (to a consult with your doctor) if you encounter a physical problem. If you feel a burning sensation or there is an exercise you don’t understand, having the help of a clinician is interesting because the clinician can help determine if the problem is related to your injury or if it is another health issue.

ISCI 05, male, 59 years

I think that it would be very useful because when you finish your rehabilitation, like myself, you feel a sort of mourning, an emptiness, and you’re all by yourself. It would be good if there was a way to provide people with the opportunity to have contact with a clinician before they finish their rehabilitation; it could be something that can be continued after.

ISCI 04, male, 68 years

However, some of the individuals who received all their rehabilitation services from the same institution preferred contact with a professional with whom they are familiar.

If you have a question, instead of calling a technician, you can come (to the rehabilitation site) and see someone who cared for you and can answer your questions, someone who knows you better than someone over the phone. Otherwise, you will need to explain [your situation] to the person you call [and...] start all over again.

ISCI 07, male, 63 years

#### Format

Participants reported that the way in which content is structured and delivered, in terms of the appearance, language, and ease of use, influences their level of interest in using a Web-based platform.

##### Appearance

The majority of participants conveyed that they are primarily visual learners, and as such, are drawn towards photos, videos, colorful diagrams, and succinct text.

Pictures, videos - I’m very visual, so they help. I don’t like text as much. So pictures, videos, and demonstrations are for sure always fun and practical.

ISCI 09, male, 19 years

Colour coding is always more interesting. It makes it less dull to read. It’s fun to at least having colour coding, which makes it more appealing to the eye. It’s more structured.

ISCI 05, male, 59 years

##### Language

Participants communicated the importance of having clear and concrete explanations that use lay and positive language. Participants also stressed upon the fact that all resources should be available in French.

[Terms such as] “muscular reinforcement” and “aerobic capacity” can be [confusing] for someone who is less knowledgeable. You know, give more concrete examples.

ISCI 10, male, 31 years

[When] it says there are English documents, I’m already less inclined to visit them because they are in English. I’m sure that the majority of our clientele (in Quebec) would feel that way

HCP 09, male, physical rehabilitation therapist, rehabilitation site 3

It seems a little negative. You know “Lack of time, I can’t participate in physical activity...” I would tend to rephrase the messages more positively. It feels very critical...We all have our reasons whether or not to participate in physical activity, good or bad. Maybe change it a little...to be more positive.

HCP 09, male, physical rehabilitation therapist, rehabilitation site 3

##### Ease of Use

Participants identified the need for a user-friendly website, comprised of structured, easily accessible content, as it renders the navigation experience more alluring and time-effective.

When you go on a website, you try to find information rapidly to accomplish something specific, [...because] we are all pressed for time. Sometimes it’s hard to find information we want on a website, so it would be good to group the information in a succinct way.

ISCI 12, male, 53 years

[It’s important] for links to be easily accessible to seek information that is relevant to me - [not to have to toggle back and forth between pages]. I would suggest using links to help quickly access relevant information.

HCP 09, male, physical rehabilitation therapist, rehabilitation site 3

#### Likelihood of Using a Web-Based Portal Tailored to the Needs of Individuals with a Spinal Cord Injury

The mean and median ratings from both groups of participants were calculated for the likelihood that they would promote and use a Web-based portal tailored to the needs of individuals with an SCI. The mean and median ratings for HCPs were 9.00 and 8.78, respectively. The mean and median ratings for individuals with an SCI were 7.75 and 7.88, respectively.

## Discussion

### Principal Findings

**Figure 1:**
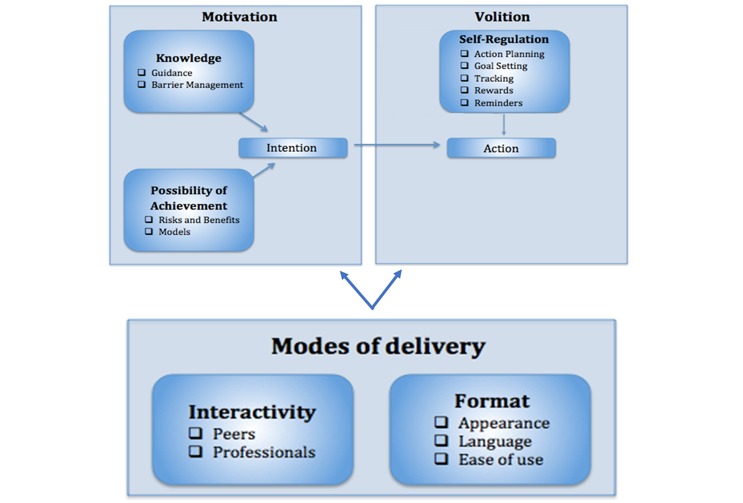
Diagram demonstrating how the resulting five themes relate to behavior change techniques and the phases of the Health Action Process Approach framework, and the how mode of delivery impacts these phases and behavior change techniques.

The aim of this study was to highlight the Web-based features that have the potential to influence the motivation and volition of individuals with an SCI to self-manage their participation in physical activity. This is the first study of its kind to use an in-depth qualitative research design to understand the needs, preferences, and lived experiences of members of the SCI community and HCPs in the preliminary stages in the development of a Web-based self-management program. Through an in-depth inductive thematic analysis, 5 core themes and 14 subthemes emerged. The first three themes (*knowledge*, *possibility of achievement*, and *self-regulation*) align well with the two phases of the Health Action Process Approach framework and thus BCTs, while the last two themes (*interactivity* and *format*) align with MoDs (Figure 1).

Tools that enhance one’s *knowledge* and convey a *possibility of achievement* can provide persons with an SCI the motivation to form an intention to participate in physical activity [46]. Participants expressed an interest in resources that offer guidance on their condition and physical activity, strategies to overcome barriers, and information on the risks and benefits. Inclusion of BCTs that improve knowledge of one’s condition has been attributed to increased levels of self-efficacy and physical activity behavior [24,25,37]. Participants also identified that *models* of individuals with an SCI successfully practicing physical activity help reinforce the intention to adopt healthy behaviors, as they contribute to a positive outlook on their condition through relatability [13]. This BCT has also been linked to improved clinical and psychological outcomes [24,47].

Participants expressed that *self-regulation* tools such as *action planning*, *goal setting*, and *tracking* are conducive to initiating and maintaining physical activity. These BCTs have demonstrated effectiveness in increasing one’s physical activity engagement and sense of control in various populations by transforming their intention into a behavior [24,25,30,47,48]. Participants’ perspectives, however, were mixed regarding the value of having *reminders* and *rewards* on a website. Although a number of the individuals and HCPs expressed that reminders have the potential to keep oneself engaged in physical activity, others perceived this function as burdensome and stressful. These findings are echoed in the literature: One systematic review demonstrated that a reminder system had the largest favorable effect on physical activity levels [36]. Conversely, Williams and French [47] associated this system with decreased self-efficacy among study participants, while a systematic review reported small and nonsignificant effects on physical activity when interventions contained reminders [24]. In terms of *rewards*, a portion of this study’s participants viewed positive reinforcements as a necessity to their physical activity practice compared to others who stated that the health benefits from regular physical activity engagement are enough of a reward on their own. Interestingly, two studies have found that a system that rewards participants based on effort or progress positively influences one’s self-confidence with respect to their physical activity participation. However, these same studies did not report significant effects where interventions rewarded participants only for successful behavior. We suggest that focusing on smaller short-term progress may increase one’s sense of self-efficacy regarding physical activity engagement [27,47,49].

Additionally, participants identified the importance of including *interactive* features on the website. For example, some participants suggested that online access to HCPs may afford individuals with an SCI a sense of reassurance and support needed to cope with setbacks in physical activity. In fact, having access to a HCP over the phone was found to increase adherence to physical activity among individuals in both the motivational and volitional phases of behavior change [26,38]. In contrast, others mentioned that online access to HCPs would not be a necessary feature, as they have sufficient contact with an HCP in their community. Participants also expressed a preference for online discussion forums where they can interact with *peers* (fellow persons with an SCI), exchange knowledge, and integrate within the active SCI community. In their study, Allin et al [44] highlighted the importance of including these community-type resources on a Web-based platform.

Furthermore, our findings reveal that the way in which information is displayed on a Web-based platform (*format*), including the *appearance*, *language*, and *ease of use*, influences the degree to which individuals are drawn to and potentially utilize Web-based resources. Recent studies have stressed the importance of addressing features and functions including usability, readability, and visual esthetics to assure end-users’ engagement in Web-based interventions [50,51]. However, research remains limited on the specific elements that constitute effective Web design [50]. More research on the impact of *format* on behavior change is needed to contribute to evidence supporting its value in online self-management portals. Ultimately, the interactive features and format of the website contribute to the degree of appeal of the BCTs identified by the first three themes and therefore have an influence on individuals’ motivation and volition to engage in physical activity.

Our research builds upon Munce and colleagues’ [15] cross-sectional study, which explored general components of self-management programs for individuals with an SCI. They reported, among others, participants’ desires for portals to be internet-based and to include a physical activity component. Although numerous Web-based self-management portals exist, website quality remains low and few incorporate theoretical frameworks, BCTs, and MoDs into their design [20,21].

The *SCI Action Canada* website is one of the most developed Web-based self-management systems that currently exists. Its design was guided by the HAPA framework and thus incorporates various BCTs and MoDs that have been linked to increased physical activity engagement among individuals with an SCI. As such, it was used as a starting point for our research, anticipating that several areas of improvement would emerge from our results, which was the case. First, the content should be updated and enhanced to reflect the needs and preferences of the SCI community. Second, interactive features, including contact with peers and professionals through the same system, should be embedded within the website. Third, while not currently incorporated into health care, introducing self-management tools to patients early on in their recovery process is an integral step to ensure a continuum of care.

Murray et al [52] found that new technology has the greatest likelihood of becoming implemented if it aligns with the organizational objectives and expertise of existing HCPs and positively influences their relationships with patients. Dwarswaard and colleagues [2] also demonstrated that individuals living with a chronic condition expect HCPs to play a vital role in their self-management, which was similarly expressed by our study subjects. Given the sudden onset of an SCI and the trajectory and intensity of rehabilitation services, embedding HCPs into this process to ensure a continuum of care is critical. This can inform the need for a self-management portal to be presented and endorsed by HCPs before discharge as well as the incorporation of interactive features on the system to ensure users feel supported in the long-term.

This study’s findings support the need to include numerous persuasive technologies in a Web-based physical activity portal. Our results align with the literature, which has found that multimode tailored interventions that incorporate video, audio, discussion forums, and chat enhance interactivity, skills, and knowledge building, thus positively influencing patient engagement and health-related outcomes [27,35,53,54]. Furthermore, previous interventions that included multiple BCTs had larger influences on behavior change than those that incorporated fewer change techniques [24].

Finally, the robust user-centered design permitted us to delve into specific Web-based components favored by members of the SCI community. As such, this study supports the need to engage persons with an SCI and HCPs early on in the design of an online self-management portal. In addition, the high ratings of these key stakeholders on the likelihood to use a website tailored to one’s needs illustrate not only the willingness of participants to use such a website, but also the degree to which it is valued in improving one’s self-management in physical activity. As such, the educational and material resources included on the proposed self-management portal should be made easily accessible and cater to individualized needs [14,38].

### Limitations

First, when considering the generalizability of this study’s findings, it is important to note that the majority of the individuals who participated (83%) reported that they have been engaging in physical activity for more than 6 months. In addition, most participants (n=8) spend at least 2 hours on the internet daily. The characteristics of our sample may be attributed to the self-selective nature of our recruitment strategy, as members of the SCI community with an interest in physical activity likely shared an interest in this study’s topic. As such, the results may not be representative of individuals with an SCI who are in an early stage of physical activity engagement or who lack access to Web-based resources. Moreover, due to our methodology and small sample size, we were unable to derive conclusive similarities or discrepancies in the findings between subjects, despite reaching saturation with 21 participants. Although this was not part of our initial objective, future research will involve conducting further analyses with more study subjects to determine subgroup clusters as we continue to develop the platform. A strength of this more engaged and technologically savvy sample, however, is that they were able to reflect on the motivational strategies they use or have used in their experience with physical activity to provide valuable feedback on Web-based persuasive technologies. These insights will guide us in shaping a platform integrated into care in a way that attracts less active individuals with an SCI to initiate behavior change and maintain engagement when discharged back to the community. Further research is needed to consider the perspectives of individuals with an SCI at a rudimentary stage of physical activity engagement.

Second, some participants reported that they did not have sufficient time to navigate the website, which may have influenced their exploration of the available resources. These participants may not have been able to conceptualize the impact of certain Web-based features they initially overlooked due to the time limit. Third, participants were asked to comment on several BCTs (eg, reward system and goal setting system) that have been shown to be effective in the literature but were not embedded within *SCI Action Canada*. Participants likely formed an opinion based on the hypothetical value of these tools without having had a genuine opportunity to interact with them on a website. Fourth, due to time and resource constraints, only persons with an SCI living in urban areas were recruited. As their perspectives may vary from those who live in more rural settings, the findings may be more applicable to an urban context.

### Conclusions

To our knowledge, this is the first study to highlight the Web-based features that should be considered in the design of a Web-based self-management platform targeting physical activity. The Health Action Process Approach, as well as BCTs and MoDs, add value to the promotion of physical activity engagement, as they shed light on the potential for Web-based features to enhance the motivation and volition of persons with an SCI. The in-depth analysis and report of the needs and preferences of both individuals with an SCI and HCPs in this study serve as an essential preliminary step in the design of an online physical activity portal. Once a prototype is created, its ability to influence adherence and behavior change will need to be evaluated. Lastly, our study contributes to the growing body of knowledge on disability and chronic disease management. Many of the challenges in the self-management of physical activity experienced by individuals living with an SCI in the self-management of physical activity are similarly experienced by members of related disability populations. As such, we highly encourage fellow researchers to utilize and build upon our robust methodology as a platform to deepen the understanding of the needs and preferences of other disability populations in the interest of promoting and enhancing quality of life. Further research is therefore warranted in this area.

## Abbreviations

BCT: behavior change technique
HAPA: Health Action Process Approach
HCP: health care professional
MoD: mode of delivery
SCI: spinal cord injury
